# 86 Outcomes for 43 Hand Burns Treated with 2:1 Meshed and Epidermal Autografts with Abundant Donors

**DOI:** 10.1093/jbcr/irac012.089

**Published:** 2022-03-23

**Authors:** Daniel Yoo, Malcolm M Taylor IV, Scott Barnett, Charles T Tuggle, Nicole Kopari, Jeffrey E Carter, Herb A Phelan

**Affiliations:** LSUHSC-New Orleans, New Orleans, Louisiana; Tulane University Department of Surgery, New Orleans, Louisiana; LSUHSC-New Orleans, New Orleans, Louisiana; LSUHSC-New Orleans, New Orleans, Louisiana; ; University Medical Center- New Orleans, New Orleans, Louisiana; LSUHSC-New Orleans Department of Surgery, New Orleans, Louisiana

## Abstract

**Introduction:**

Our group has previously reported our experience in treating hand burns with 2:1 meshed autografting and simultaneous application of autologous skin cell suspension (ASCS) even when donor sites are abundant. Here, we sought to expand on this experience. We hypothesized that the use of 2:1 meshed autografting and ASCS (MA/ASCS) would provide comparable outcomes to hand burns treated with sheet graft.

**Methods:**

A retrospective review was conducted of all subjects operated on for deep 2^nd^ and 3^rd^ degree hand burns at our ABA-verified burn center from April, 2018 to May, 2021. Exclusion criterion was a burn of >20% TBSA. The cohorts were those subjects treated with MA/ASCS versus those treated with split thickness sheet, pie-crust, or 1:1 meshed autograft alone (STAG). Outcomes included proportion returning to work (RTW), length of time for RTW, and time to wound closure. Mann-Whitney U test was used for comparisons of continuous variables, and Fishers Exact test for categorical variables. Values are reported as median and interquartile range.

**Results:**

Sixty-eight subjects fit the study criteria (MA/ASCS n=43, STAG n=25). The MA/ASCS group was significantly older than the STAG cohort (45.5 yrs [32, 59.25] vs 35 [28, 45], p=0.013) with larger %TBSA burns overall (11.5% [7, 16.25] vs 2% [1, 3], p < 0.0001), and larger hand burns (186 cm2 [124.75, 330.5] vs 104 cm2 [56, 164], p=0.001). Comparable results were seen between MA/ASCS and STAG, respectively, for time to wound closure (8 days [7, 13] vs 8 [6, 14], p=0.48), proportion RTW (51% vs 64%, p=0.33), and days for RTW among those returning (38 [29.25, 62.75] vs 34.5 [20.75, 57], p=0.471). Fractional ablations were performed in 14% of the MA/ASCS group and 12% of the STAG group.

**Conclusions:**

Despite being significantly older, having larger hand wounds, and larger overall wounds within the parameters of the study criteria, patients with 20% TBSA burns or smaller whose hand burns were treated with 2:1 mesh and ASCS overspray had comparable time to wound closure and return to work as subjects treated with 1:1, pie-crust, or sheet STAG.

